# Time from Symptom Onset to Hospitalisation of Coronavirus Disease 2019 (COVID-19) Cases: Implications for the Proportion of Transmissions from Infectors with Few Symptoms

**DOI:** 10.3390/jcm9051297

**Published:** 2020-05-01

**Authors:** Robin N. Thompson, Francesca A. Lovell-Read, Uri Obolski

**Affiliations:** 1 Mathematical Institute, University of Oxford, Oxford OX2 6GG, UK; francesca.lovell-read@merton.ox.ac.uk; 2 Christ Church, University of Oxford, Oxford OX1 1DP, UK; 3 School of Public Health, Tel Aviv University, Tel Aviv 6997801, Israel; 4 Porter School of the Environment and Earth Sciences, Tel Aviv University, Tel Aviv 6997801, Israel

**Keywords:** COVID-19, symptoms, infectiousness, presymptomatic transmission, infectious period, symptom onset to hospitalisation, reproduction number

## Abstract

Interventions targeting symptomatic hosts and their contacts were successful in bringing the 2003 SARS pandemic under control. In contrast, the COVID-19 pandemic has been harder to contain, partly because of its wide spectrum of symptoms in infectious hosts. Current evidence suggests that individuals can transmit the novel coronavirus while displaying few symptoms. Here, we show that the proportion of infections arising from hosts with few symptoms at the start of an outbreak can, in combination with the basic reproduction number, indicate whether or not interventions targeting symptomatic hosts are likely to be effective. However, as an outbreak continues, the proportion of infections arising from hosts with few symptoms changes in response to control measures. A high proportion of infections from hosts with few symptoms after the initial stages of an outbreak is only problematic if the rate of new infections remains high. Otherwise, it can simply indicate that symptomatic transmissions are being prevented successfully. This should be considered when interpreting estimates of the extent of transmission from hosts with few COVID-19 symptoms.

## 1. Introduction

The coronavirus disease 2019 (COVID-19) pandemic continues to pose a significant threat to public health, with more than three million cases reported globally, including over 228,000 deaths (as of 30 April 2020). The causal agent is severe acute respiratory syndrome coronavirus 2 (SARS-CoV-2), and extensive public health measures are being deployed around the world to mitigate the spread of this virus [1,2,3,4,5,6,7,8,9]. In the United Kingdom, initial interventions focused on preventing transmission from the individuals that were most likely to be infected [10]. For example, symptomatic individuals and their contacts, as well as anyone who had visited severely affected areas, were asked to self-isolate. Since these measures were not sufficient to prevent sustained transmission, interventions have now intensified, with all members of the public being asked to stay at home. Interventions are therefore no longer targeted specifically at individuals with a higher risk of having contracted the virus. 

**Figure 1:**
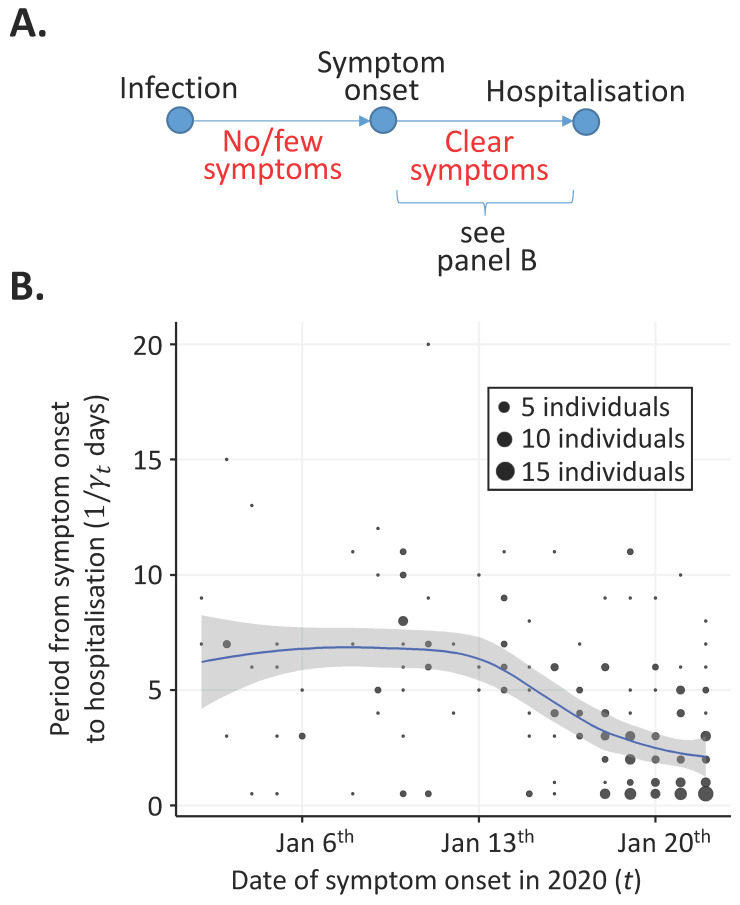
Changes in the period between symptom onset and hospitalisation (the assumed symptomatic infectious period) from 2 January 2020 to 22 January 2020. (**A**) Schematic showing the assumed epidemiology of infected hosts. Infected individuals initially have no or few symptoms. Later in infection, infected individuals develop clear symptoms. (**B**) Estimated mean period between symptom onset and hospitalisation (blue), along with the corresponding 95% confidence interval for the mean value (grey shaded region). Circle areas are proportional to the numbers of individuals with symptom onset date *t* who were hospitalised $1/\gamma_{t}$ days later.

The routine public health measures that were implemented early in the COVID-19 pandemic have been successful in the past. For example, the 2003 SARS pandemic was eventually brought under control following a policy based on detection and isolation of symptomatic hosts and their contacts [11]. One of the key factors determining the success of such measures during any infectious disease outbreak is the level of symptoms exhibited by individuals who are transmitting the pathogen [12,13]. The proportion of transmissions occurring prior to symptoms has been noted as particularly important [14]. This is because individuals displaying few symptoms are harder to detect and isolate or treat effectively than those with clear, disease-specific symptoms. After infection by SARS-CoV-2, hosts develop mild symptoms initially, such as a dry cough and fever, followed by more serious symptoms, such as breathing difficulties (Figure 1A, see also [15,16]). Initial symptoms tend not to be specific to COVID-19, although, for a limited number of patients, gastrointestinal symptoms might be an early and distinct indicator of infection by SARS-CoV-2 [17].

Evidence is slowly accumulating about the relationship between COVID-19 symptoms and the transmissibility of SARS-CoV-2 [14,18,19,20,21]. It has become increasingly apparent that individuals can transmit the virus when they are not displaying clear symptoms [22,23,24,25,26,27]. A recent study by Ferretti et al. [14] distinguished between: (i) Symptomatic transmissions (direct transmissions from individuals displaying clear symptoms); (ii) Presymptomatic transmissions (direct transmissions before the source individual develops clear symptoms) and (iii) Asymptomatic transmissions (direct transmissions from individuals who never develop clear symptoms). In that study, one of the key findings was that SARS-CoV-2 spreads too fast for containment by routine public health measures such as standard contact tracing and isolation of known infected hosts. This is partly because of the significant proportion of transmissions occurring prior to clear symptoms developing.

Here, we consider the earliest stages of the COVID-19 pandemic and show that the proportion of infections arising from individuals with few symptoms (including infected individuals with no symptoms at all) is a quantity that changes during an outbreak. In our initial analysis, we consider the control of symptomatic hosts and estimate changes in the time period between symptom onset and hospitalisation using data for 212 patients who reported symptoms between 2 January 2020 and 22 January 2020. The period between symptom onset and hospitalisation reflects the time in which symptomatic hosts were potentially transmitting the virus in the community and is a proxy for the time period between individuals developing clear symptoms and being isolated.

Then, we turn our attention to infected individuals with few symptoms. The proportion of transmissions occurring from hosts with few symptoms at the start of any outbreak is an important factor affecting the controllability and severity of the outbreak. For a fixed value of the basic reproduction number, there is a threshold value of the initial proportion of transmissions occurring from hosts with few symptoms. If the initial proportion of transmissions occurring from hosts with few symptoms is above this threshold, then the outbreak cannot be controlled by interventions targeting hosts exhibiting clear symptoms alone.

However, as public health measures and public awareness have changed during the COVID-19 pandemic, the proportion of transmissions from hosts with few symptoms will have varied. As we show, changes in the period between symptom onset and hospitalisation lead to temporal variations in the proportion of transmissions occurring from hosts prior to clear symptom development. Consequently, after the start of the pandemic, a high proportion of transmissions from individuals with few symptoms is not always indicative of an uncontrollable outbreak. Instead, it can show that some transmissions from infected individuals with clear symptoms are being prevented effectively.

## 2. Methods

### 2.1. Time from Symptom Onset to Hospitalisation

We considered temporal changes in the time period from symptom onset to hospitalisation during the initial stages of the COVID-19 pandemic. We denote the expected time from symptom onset to hospitalisation for an individual developing clear symptoms on day *t* by $1/\gamma_{t}$ days, where $\gamma_{t}$ is the rate of hospitalisation. In our analysis, the value *t* = 0 days corresponds to 2 January 2020.

We estimated $1/\gamma_{t}$ using a publicly available line list dataset describing this period for 231 patients from around the world [28]. Implausible time periods (negative values and periods longer than 30 days) were assumed to be reported incorrectly and were excluded from the analysis. There were 19 such patients, so that our analysis was performed with data from 212 patients (Supplementary Material: Table S1). Of these, 190 patients were from China and 22 were from other countries.

We used data for patients with dates of symptom onset up to 22 January 2020 (i.e., *t* = 20 days). This is because, when the analysis was performed (on 9 February 2020, i.e., 18 days later), it is unlikely that individuals symptomatic on 22 January 2020 would not yet have been hospitalised. In the data used in our analysis, only one patient was recorded as having a period between symptom onset and hospitalisation longer than 18 days. If we had considered data from patients with later symptom onset dates than 22 January 2020, then “right censoring” may have occurred [29,30,31]. Specifically, we could have been preferentially including patients with short periods between symptom onset and hospitalisation in the analysis, leading to underestimation of the symptomatic infectious period (i.e., the part of the infectious period during which individuals display clear symptoms).

The symptomatic infectious period was assumed to be the difference between the symptom onset and hospitalisation dates. When symptom onset and hospitalisation occurred on the same day, we assumed that the period between these times was equal to 0.5 days rather than 0 days, since immediate hospitalisation is impossible. Locally estimated scatterplot smoothing (LOESS) with a span parameter of 0.75 and tricubic distance weighting (the default settings in the R software package STATS) was used to estimate $1/\gamma_{t}$. All analyses in this manuscript were performed using R software version 3.6.1.

### 2.2. Time-Varying Reproduction Number

We define the time-varying reproduction number, $R_{t}$, as the expected number of secondary infections over their entire course of infection generated by an individual who first shows clear symptoms on day t (where t=0 corresponds to 2 January 2020) [32,33,34,35,36]. Over the course of a coronavirus infection, infected hosts tend to have few symptoms initially, before potentially developing more clear symptoms (Figure 1A). The time-varying reproduction number (which we refer to as “the reproduction number” hereafter) is the sum of the expected number of secondary infections when the individual has few symptoms and the expected number of secondary infections when that individual has clear symptoms. The basic reproduction number, $R_{0}$, corresponds to the reproduction number at time t=0.

In our analysis, we considered changes in the reproduction number due to temporal variations in the time between symptom onset and hospitalisation alone. The expected number of infections arising from the infectious period with few symptoms was assumed unchanged during the outbreak, and is given by $\alpha_{0}R_{0}$, where $\alpha_{0}$ represents the expected proportion of infections generated by infectious hosts in the period before clear symptoms near the beginning of the outbreak (i.e., *t* = 0). The expected number of infections arising from the symptomatic infectious period is then given by $\left(1-\alpha_{0}\right)R_{0}\left(\frac{1/\gamma_{t}}{1/\gamma_{0}}\right)$, where the factor $\left(\frac{1/\gamma_{t}}{1/\gamma_{0}}\right)$ reflects the change in the symptomatic infectious period between the beginning of the period considered and time *t*. Consequently, the reproduction number is given by

(1) $$R_{t}=\alpha_{0}R_{0}+\left(1-\alpha_{0}\right)R_{0}\left(\frac{1/\gamma_{t}}{1/\gamma_{0}}\right)$$

### 2.3. Proportion of Transmissions from Individuals with Few Symptoms

The proportion of transmissions occurring from infectious hosts with few symptoms (i.e., the expected proportion of secondary infections generated by an infected host that arise before that host develops clear symptoms) is a dynamic quantity that changes throughout the outbreak. We denote this quantity by $\alpha_{t}$. An expression for $\alpha_{t}$ is derived by considering the ratio between the expected number of transmissions in the infectious period when there are few symptoms and the reproduction number:

(2) $$\alpha_{t}=\frac{\alpha_{0}R_{0}}{\alpha_{0}R_{0}+\left(1-\alpha_{0}\right)R_{0}\left(\frac{1/\gamma_{t}}{1/\gamma_{0}}\right)}=\frac{\alpha_{0}}{\alpha_{0}+\left(1-\alpha_{0}\right)\left(\frac{1/\gamma_{t}}{1/\gamma_{0}}\right)}$$

We note that this final expression for $\alpha_{t}$ does not depend on the value of the basic reproduction number, $R_{0}$.

## 3. Results

### 3.1. Time from Symptom Onset to Hospitalisation

As described in the Methods, we considered how the time from symptom onset to hospitalisation changed in the initial stages of the COVID-19 pandemic (Figure 1B). The estimated mean value of this quantity changed from around 6.5 days in the period between 2 January and 14 January to a lower value of around 2 days by 22 January. Towards the end of the time period considered, the mean time from symptom onset to hospitalisation appeared to be reducing more slowly than earlier in the time period considered. This potentially indicates a limit to the ability of isolation measures to decrease the symptomatic infectious period.

### 3.2. Reproduction Number and Proportion of Transmissions from Individuals with Few Symptoms

If the period from symptom onset to hospitalisation tends to decrease during an outbreak, the expected number (and proportion) of transmissions from hosts displaying clear symptoms decreases. In that case, the reproduction number can be expected to decrease, and the relative proportion of transmissions from individuals with few symptoms will increase.

Of particular interest is the threshold for outbreak control, i.e., $R_{t}=1$ [32,35]. For a single value of $R_{0}$, there is a corresponding threshold value of $\alpha_{0}$ that determines whether or not the outbreak can ever be brought under control via the isolation of symptomatic hosts alone. In particular, in the impossible case in which symptomatic hosts are isolated perfectly and immediately, so that $1/\gamma_{t}$ approaches zero days, then Equation (1) indicates that the threshold for outbreak control is achieved exactly if $\alpha_{0}=\frac{1}{R_{0}}$. Consequently, if $\alpha_{0}>\frac{1}{R_{0}}$, then in the absence of other effects (e.g., acquired immunity as the pathogen infects individuals in the population), any intervention that only involves control of hosts with clear symptoms will fail to bring $R_{t}<1$ and therefore will not bring the outbreak under control.

**Figure 2:**
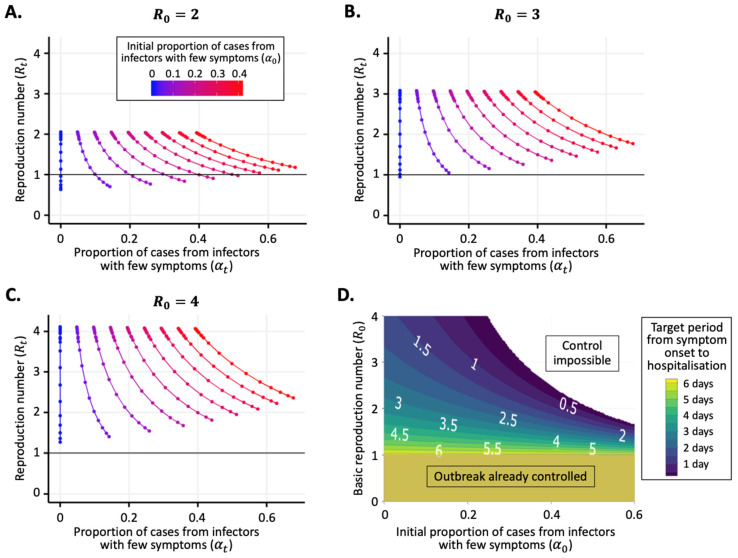
The reproduction number ($R_{t}$ ) and the proportion of transmissions from hosts with few symptoms ($\alpha_{t}$) vary in response to changes in the period between symptom onset and hospitalisation. (**A**) Variation in $R_{t}$ and $\alpha_{t}$ between 2 January and 22 January 2020 due to changes in the mean time from symptom onset to hospitalisation ($1/\gamma_{t}$ days; see Figure 1B), under the assumption that $R_{0}=2$. Values of $R_{t}$ and $\alpha_{t}$ were calculated using Equations (1) and (2), respectively. Lines represent different values of the initial proportion of transmissions from hosts with few symptoms ($\alpha_{0}$). Values of $\alpha_{0}$ between 0 and 0.4 were considered in steps of 0.05 (i.e., nine values in total). In the period from 2 January to 22 January 2020, transmissions from hosts with clear symptoms were typically prevented increasingly effectively, leading to a temporal trend from the tops to the bottoms of the lines shown. (**B**) Equivalent figure to panel A, but with $R_{0}=3$. (**C**) Equivalent figure to panel A, but with $R_{0}=4$. (**D**) Required time within which symptomatic infectious hosts must be isolated on average ($1/\gamma_{t}$ days) so that $R_{t}$ is less than one (i.e., the outbreak is controlled), calculated using Equation (1) for different pairs of values of $\alpha_{0}$ and $R_{0}$. In panels A-C, the horizontal black line shows the threshold value of $R_{t}$ for outbreak control ($R_{t}=1$). The value of $1/\gamma_{0}$ used in all panels is 6.7 days (as estimated in Figure 1B).

Since perfect control of symptomatic infectious hosts is impossible to achieve in reality, some values of $\alpha_{0}$ that are less than $\frac{1}{R_{0}}$ are also likely to correspond to scenarios in which isolation of symptomatic hosts alone will fail to control the outbreak. We explored this in the specific context of the range of symptomatic infectious periods shown in Figure 1B. For example, when $R_{0}=2$ (Figure 2A), then any value for the proportion of transmissions from hosts with few symptoms on 2 January below $\alpha_{0}=0.27$ will have seen $R_{t}$ reduce below 1 by 22 January 2020. If, instead, $R_{0}=3$ (Figure 2B), then isolation of hosts with clear symptoms was only able to reduce $R_{t}$ below 1 by 22 January 2020 if the proportion of transmissions from hosts with few symptoms was very low (specifically $\alpha_{0}<0.03$). For values of $R_{0}>3.2$, even if no transmissions occur from hosts with few symptoms, then $R_{t}$ would not have been reduced below 1 by 22 January 2020 given the reduction in the symptomatic infectious period shown in Figure 1B.

If transmissions from hosts with clear symptoms could be eliminated completely, then it might or might not have been possible to reduce $R_{t}$ below 1 without resorting to the current “lockdowns” in place in countries worldwide, depending on the precise values of $R_{0}$ and $\alpha_{0}$. In Figure 2D, we show the target period from symptom onset to hospitalisation (i.e., the mean value of this period below which $R_{t}$ is less than one) for pairs of values of $R_{0}$ and $\alpha_{0}$ (assuming that $1/\gamma_{0}=6.7$ days, as in Figure 1B). The calculation of the target period from symptom onset to hospitalisation for each pair of values simply involved rearranging Equation (1) to find the critical value of $1/\gamma_{t}$ days corresponding to $R_{t}=1$. If $R_{0}<1$, then the outbreak was already under control when t=0 days and so there was no target period from symptom onset to hospitalisation (bottom of Figure 2D). If $\alpha_{0}$ and $R_{0}$ were both large, then it would be impossible to bring $R_{t}<1$ by reducing the symptomatic infectious period alone (top right of Figure 2D). In general, if $\alpha_{0}$ and $R_{0}$ were larger, then the target period from symptom onset to hospitalisation for outbreak control would be shorter.

## 4. Discussion

In this article, we used data from early in the COVID-19 pandemic to demonstrate that the proportion of transmissions arising from infectors with few symptoms changes during an outbreak. We found that, in the initial stage of the pandemic, symptomatic hosts were hospitalised/isolated increasingly effectively (Figure 1B). This is likely to be due to changes in public awareness about the outbreak, as well as the introduction of containment measures (e.g., contact tracing followed by isolation of secondary infected hosts). The consequences of improved isolation of symptomatic hosts, in the absence of changes to other factors, are that the proportion of infections due to hosts with few symptoms is likely to have increased and the reproduction number is likely to have decreased (Figure 2A–C).

We considered the impact of transmissions from infectors with few symptoms on the controllability of infectious disease outbreaks, using COVID-19 as a case study. If symptomatic hosts can be isolated effectively, then it may be possible to bring an outbreak under control (Figure 2A). However, this depends on the value of the basic reproduction number ($R_{0}$) and the initial proportion of infections arising from hosts with few symptoms ($\alpha_{0}$). If the values of $R_{0}$ and $\alpha_{0}$ are both high, then control of any outbreak by targeting symptomatic hosts alone is impossible (top right region of Figure 2D). Under this scenario, other public health measures are necessary [13], for example, strategies involving finding and isolating hosts with few symptoms (e.g., tracing and testing of known contacts, whether or not they are showing clear symptoms [2]) or reducing transmission via large-scale interventions that are not focused on infectious hosts alone (e.g., school closures, workplace closures, prevention of large-scale gatherings and/or transport bans, most of which are now in place for COVID-19 in a number of countries worldwide [37]).

We also found that temporal variations in the proportion of transmissions occurring from hosts with few symptoms are independent of the value of $R_{0}$. As an example, if the initial percentage of transmissions from individuals with few symptoms is 10%, then Equation (2) indicates that a 50% reduction in the time from symptom onset to hospitalisation would correspond to the proportion of transmissions from hosts with few symptoms increasing to 18%, regardless of the value of $R_{0}$.

In this study, we developed a simple model that includes both transmissions from infected individuals with few symptoms and transmissions from infected individuals with clear symptoms. Our main goal was not to estimate temporal changes in $R_{t}$ and $\alpha_{t}$ during the COVID-19 pandemic accounting for the wide range of factors affecting these quantities. This would require analyses of different datasets, and it is challenging to disentangle the effects of the many factors that affect transmissibility [32,38,39]. Instead, we considered the specific effect that increasingly efficient isolation of symptomatic hosts alone has on the values of $R_{t}$ and $\alpha_{t}$. We therefore focused on changes in the symptomatic infectious period. However, the model could be extended to account for additional features of emerging outbreaks. As an example, precisely what constitutes “few symptoms” and “clear symptoms” might change as an outbreak is ongoing. For COVID-19, initial symptoms are nonspecific. Consequently, when case numbers were very low early in the pandemic, an individual developing such symptoms may not have considered them to be indicative of infection by SARS-CoV-2. Now that the number of cases has increased, an individual might assume that nonspecific symptoms are an indicator of infection by SARS-CoV-2 and might self-isolate despite not having developed more obvious symptoms. Our assumption that the expected number of infections in the infectious period with few symptoms is unchanged during an outbreak could be amended to reflect this.

As in previous studies, we assumed that the period between symptom onset and hospitalisation is a proxy for the period between symptom onset and isolation [1,4]. We could have made other assumptions about the symptomatic infectious period—for example, assuming that an infected host is isolated at the point that the SARS-CoV-2 infection is laboratory confirmed rather than at the time of the first medical visit. Whereas this would lead to a longer symptomatic infectious period than the value estimated here, the opposite effect would be seen if individuals that self-isolated at home early in their infection were included in the analysis. We also used a simple method to estimate the mean length of the period between symptom onset and hospitalisation, namely LOESS. One advantage of this approach is that it did not require a specific distribution for the time from symptom onset to hospitalisation to be chosen. However, we did assume that the period between symptom onset and hospitalisation was 0.5 days for individuals that developed symptoms and were hospitalised on the same day. In theory, other methods of parameter estimation could be used. For example, the period between symptom onset and hospitalisation could be estimated using Markov chain Monte Carlo in shifting time windows throughout the outbreak. This would have the advantage that interval-censored data could be specified for possible periods between symptom onset and hospitalisation for each host in the dataset, rather than assuming a precise value (see, e.g., [1,32,40]). However, this would involve assuming a parametric distribution (or range of distributions) characterising the symptomatic infectious period, which was not necessary in our analysis.

Despite these simplifications, our analysis has allowed us to demonstrate the principle that the proportion of transmissions from individuals with few symptoms is not a fixed quantity and has instead varied temporally during the COVID-19 pandemic so far. Such changes are particularly likely early in an outbreak and whenever intervention strategies are altered. The proportion of transmissions from individuals with few symptoms at the beginning of any outbreak can, in theory, be used, along with the value of the basic reproduction number, to predict whether or not an outbreak can be brought under control via isolation of symptomatic hosts alone. If these quantities are known simultaneously at another point in time, a similar assessment can be performed. Nonetheless, whenever control of symptomatic hosts is enhanced, the proportion of transmissions from individuals with few symptoms is expected to increase.

Since we began our analysis, a range of values have been estimated for the proportion of transmissions occurring from individuals with few symptoms for COVID-19 [14,41,42]. For example, Ferretti et al. [14] estimated a range of between one-third and one-half of transmissions occurring from presymptomatic infected individuals using data from 40 source-recipient pairs. As our results show, estimates of the proportion of transmissions occurring from individuals with few symptoms alone should not be used to assess whether or not an outbreak is controllable. Instead, a thorough investigation, like the one conducted by Ferretti et al. [14], is required to make that assessment. The proportion of transmissions occurring from individuals with few symptoms might take a high value as a result of measures that have reduced transmission from hosts with clear symptoms. This should be considered when interpreting estimates of the proportion of infections arising from individuals with few symptoms during the COVID-19 pandemic.

## Supplementary Materials

The supplementary material is available online at https://www.mdpi.com/2077-0383/9/5/1297/s1. Table S1, Data.
